# How Do Donor-Recipient CMV Serostatus and Post-Hematopoietic Stem Cell Transplantation CMV Reactivation Affect Outcomes in Acute Leukemia Patients?

**Published:** 2017-07-01

**Authors:** Mohammad Vaezi, Amir Kasaeian, Maryam Souri, Faeze Soufiyan, Amir Shokri Boosjin, Seyed Amin Setarehdan, Kamran Alimoghaddam, Ardeshir Ghavamzadeh

**Affiliations:** Hematology-Oncology and Stem Cell Transplantation Research Center, Tehran University of Medical Sciences, Tehran, Iran

**Keywords:** CMV serostatus, Hematopoietic stem cell transplantation, Outcome, CMV infection, Acute leukemia

## Abstract

**Background**: This study evaluated CMV serostatus in donors and recipients of hematopoietic stem cell transplantation (HSCT) and its effects on CMV reactivation of patients and all aspects of CMV on HSCT outcomes.

**Materials** **and Methods**: Seven hundred and five adult acute leukemia patients (AML=408 and AML=297) who had undergone HSCT were included in this retrospective study. We categorized donor-recipient pairs in three risk groups: positive donors (D+) were studied as high-risk group, including either R+ or R-(n=485), R-D- as low-risk group (n=32) and R+D- as intermediate group (n=15).

**Results:** There was no statistically difference in CMV reactivation among these risk groups (P=0.14).CMV infection rate was lower in R+D+ than R+D-(p=0.050). Multivariate analysis showed that patients developing CMV infection had lower overall survival (p=0.04, HR: 1.43, CI=1.00- 2.05) and higher non- relapse mortality (P=0.01, HR: 1.62, CI=1.11-2.38). Relapse rate did not change in CMV reactivated patients (P=0.94).

**Conclusion:** The results of the study indicated that asCMV reactivation occurred more in R+D- patients compared to R+D+ ones, and was associated with inferior OS and higher NRM it could be suggested that in contrast to general belief, if the recipient is seropositive , seropositive donor is preferred to a seronegative one.

## Introduction

 The relationship between CMV infection and disease relapse after hematopoietic stem cell transplantation (HSCT) has long been a subject of debate. For the first time Lönnqvist et al, cohort study showed that patients with CMV infection had less relapse compared with those withoutinfection^1^. Some Other studies have reported this association between donors or recipient CMV serostatus and reduced risk of relapseafterwards^2^^-^^4^. Yet there are several studies that did not confirm this relation^5^^-^^9^. According to recent studies, early CMV reactivation in acute myeloid leukemia (AML), except in other hematological malignancies, adults and children may be associated with a reduced risk of post- transplant relapse^10^^-^^11^. The exact mechanism through which CMV affects disease relapse after transplantation is not obvious, but it might be due to activation of cytotoxic lymphocytes or natural killer cells which attack malignant cells^12^. Besides the well-established relationship between acute GvHD and post-transplant CMV reactivation, it is supposed that chronic GvHD may occur as a result of immunity reactivation of donor cells against CMV infected cells of recipient^13^^-^^14^.

 Prognostic effects of CMV serostatus of donor-recipient pairs on transplant outcomes are also an issue of controversy. There are studies showing that CMV seronegativity of both donor and recipient is associated with lower transplant mortality.^15^Ljungman et al. study reported that both donor and recipient seropositivity favor HSCT outcome only in transplantation with unrelated donor.^6^ Some other studies have reported different results. To add information to this conflicting era, in this study,we evaluated post-allogeneic transplant outcomes, including overall survival and relapse in acute leukemia patients regarding to CMV serostatus of donor and recipient and post-transplant CMV reactivation.

## MATERIALS AND METHODS

 Adult acute leukemia patients who had undergone HSCT in our center during 2008-2014 were included in this retrospective study. Once we obtained signed informed consent from study participants, we reviewed their medical profiles.Demographic, clinical, and laboratory data of patients and donors were collected from their medical profiles using a checklist.

Conditioning regimen was non-TBI (total body irradiation) including oral Busulfan 4mg/kg, from day-6 to day-3 and Cyclophosphamide 60 mg/kg on day-2 and day-1. Stem cell source in all patients was peripheral blood. Before transplantation, CMV serostatus was evaluated in all donors and recipients by Enzyme-linked immunosorbent assay (ELISA). Recipients from matched unrelated, other related and haploidentical donors received ATG (antithymocyte globulin) immediately before transplantation for two and three days, respectively. Cyclosporine and Methotrexate were used for Graft-versus-host disease (GVHD) prophylaxis.

CMV evaluation was done by PCR technique twice weekly from transplantation till recovery or engraftment time and then CMV pp65 antigenemia was examined weekly up to 100 days after transplantation. The test was then performed in cases of clinical suspicion of CMV. Treatment was started as soon as CMV PCR was positive or 3-5 PP65 -antigen–positive cells were detected in 50,000 white blood cells or when titer was 1 pp65 + cell/50,000 white cells with GvHD and also in symptomatic patients. Ganciclovir (5mg/kg/dose) was administered twice daily until CMV test proved negative. Drug was continued for one week in half dose and then discontinued. Bone marrow morphology and chimerism analysis were done on days +15, +30, +60, +90 post-transplant and whenever it seems necessary. In every visit, patients were evaluated regarding to GvHD, and, if necessary, appropriate treatments were given.

**Outcomes and definitions**

The outcomes of this study were overall survival (OS), relapse-free survival (RFS), relapse, engraftment, acute GvHD (aGvHD), chronic GvHD (cGvHD) and non-relapse mortality (NRM). OS was the time between HSCT to death, regardless of the cause. RFS was the length of time after transplantation during which no disease was found. Relapse was determined by presence of >5% BM blasts and/or reappearance of the underlying disease. Engraftment was determined by recovery of neutrophil and platelet. Neutrophil recovery was defined as ANC (absolute neutrophil count) ≥ 500cells/µL in three consecutive days. Platelet recovery was platelet count≥ 20000 cells/µL. NRM was determined as death due to causes unrelated to disease relapse. Acute and chronic GvHD were graded according to published criteria^16^. In this survey, patients with aGvHD≥ grade II were taken into account.

Patients were divided into three risk groups according to pre-transplant serostatus of donors and recipients as follows: high risk: recipient + and donor + (R+D+) or recipient – and donor +(R–D+)*, *intermediate risk: recipient+ and donor–(R+D–) and low risk: recipient – and D–(R–D–).

**Statistical analysis**

Patients followed beyond 5 years were censored to better compare different groups as serious differences between follow-up periods can seriously bias the findings. Median follow-up time was computed by the reverse Kaplan-Meier method.^17^OS and RFS rates were estimated by the Kaplan-Meier method and compared among different categories of each covariate, using the log-rank χ² test.^18^Univariate and multivariate analyses of OS and RFS in order to calculate the hazard ratio (HR) between different categories of each covariate were performed, using a Cox proportional hazard regression^19^. The assumption of proportionality of hazards was tested for each covariate, using Schoenfeld’s residuals and plotting criteria. Gray’s method ^20^was used to calculate cumulative incidences of relapse and NRM. Death without relapse was considered as a competing event for relapse, and relapse was considered as a competing event for NRM. Fine-Gray proportional hazard regression model applied to test the effects of covariates on relapse incidence and NRM .^21^ All the variables with a P-value at or below 0.2 in the univariate Cox proportional hazard regression and the univariate Fine-Gray proportional hazard regression were included in the corresponding multivariate analyses. A significance level of 0.05 was used for all analyses. Stata (version 11.2, Stata Corp LP, College Station, TX, USA), survival package and cmprskpackage in R software version 3.2.2 were used to conduct the analyses .^22^

## Results

 Of 705 patients with acute leukemia, 408(58%) had acute myeloblasticleukemia (AML) and 297(42%) had acute lymphoblastic leukemia (ALL). Six hundred and sixty (N=660, 93.62%) patients received transplantation from fully-matched sibling donors, 24(3.4%)and 21(2.98%) of whom receivedtransplantation from matched unrelated and mismatched related donors, respectively. Source of transplantation was peripheral blood in all patients. Four hundred and twelve (N=412, 58.44%) of recipients were male and 293(41.56%) of whom were female. Mean age of patients was 25.7 years. Two hundred and eight (N=208, 29.5%) patients died during the study. The most common cause of death was relapse (67.28%) and the second one was GvHD (15.21%).Median time to engraftment was 12 days; and likewise, the median time to ANC engraftment was 12 days. The median time to platelet engraftment was 16 days. Median follow-up time was 3.8 years. Totally, relapse occurred in 27.09% of all patients.

**CMV serostatus, reactivation and outcomes**

As detected by pre-transplant ELISA test, 92.48% of recipients were serologically positive for CMV IgGand 91.17% of donors were CMV IgG seropositive. Thirty-two (6%) transplantations were low risk according to CMV serostatus of donors and recipients (R-D-). In 477 (89.66%) transplantations, both donors and recipients were seropositive (R+D+), and in 8 (1.5%) transplantations, recipients with negative CMV antibody received stem cells from donors with positive CMV antibody(R-D+).As mentioned above, these two later groups were considered as high risk. The rest of 15 (2.82%) cases were R+D-(intermediate risk). 

**CMV reactivation**: Median time to CMV reactivation was 49 days. CMV infection (reactivation) occurred in 224 of 532 (42.1%) patients evaluated as follows: 13/32(40.62%) of R-D-, 4/8(50%) of R-D+, 197/477(41.2%) of R+D+ and 10/15 (66.6%) of R+D-. CMV reactivation was not statistically associated with the risk group (P=0.14).Likewise, seronegative status of recipients did not affect the likelihood of CMV reactivation (P=0.631). Seropositive status of donors did affect the likelihood of CMV reactivation as CMV infection rate was lower in seropositive (R+D+) recipients with seropositive donors than seropositive recipients with seronegative donors (R+D-) (P=0.050).

**Overall survival**: CMV infection did not change the OS (P=0.163, Fig. 1a). 

**Figure 1:**
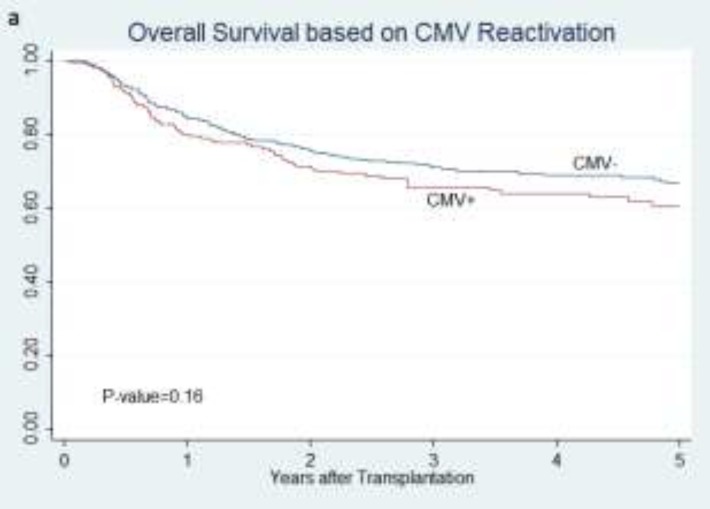
Overall survival of all patients according to CMV reactivation status (a),

Overall survival was statistically different among pre-transplant risk groups (P=0.033), and it was better in patients of high-risk group, but among high-risk patients (R+D+ and R-D+) no significant difference was observed. Seropositive recipients had better OS (P=0.017), but there was no difference between OS of R+D- and R+D+ (P=0.08). Besides, donor seropositivilty improved OS (P=0.005). No significant difference was observed in OS between R-D+ and R-D- groups (P=0.74).OS of R-D- group was statistically lower than other states altogether (P=0.02). Regarding time of CMV reactivation, no significant difference was found in OS between patients developing CMV infection before and after day+30 (P=0.57). Similarly, there was no difference in overall survival rate between reactivation before and after 100^th^ day (P=0.40).

**RFS:** CMV reactivation did not change RFS (P=0.25, Fig.1b). 

**Figure 1:**
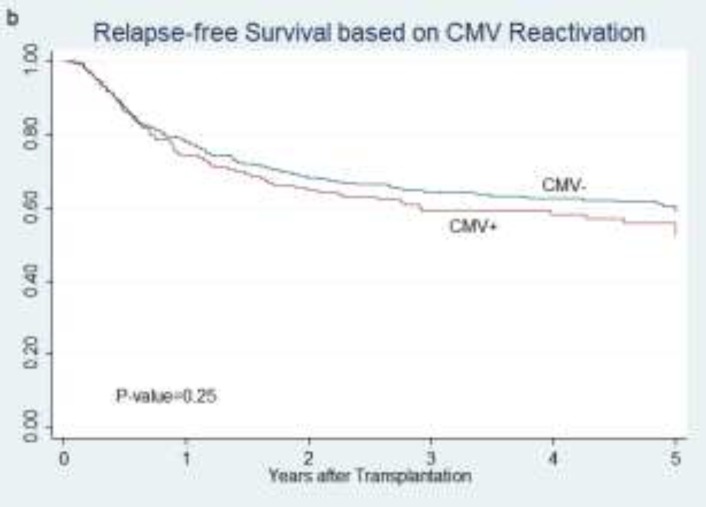
relapse-free survival of all patients according to CMV reactivation status (b)

CMV infection before and after days +30 and +100 had no different RFSS (P=0.55 and P=0.81, respectively). Recipient CMV status did not influence on RFS (P=0.055). RFS was not statistically different between R+D+ and R+D- groups (P=0.15) as well as R-D- and R-D+ groups (P=0.54).Donor seropositivity, regardless of recipients’ serostatus, improved RFS (P=0.014). No significant difference was noted in RFS among three risk-groups (P=0.049) because RFS in high-risk group patients was better than other two groups (P=0.014). On the other hand, transplantation from seronegative donor to seronegative recipient(R-D-) decreased RFS compared to other states altogether (P=0.04). In patients with CMV reactivation, positive history of CMV did not influence RFS as well (P=0.65).

**ANC and Plt engraftment** *:* ANC and Plt engraftment rates before day+30 were higher in pre-transplant low-risk group (P=0.002 and 0.004, respectively). Engraftment of ANC and Plt before day +30 were not different in patients who developed CMV infection compared with those who didn’t (P=0.2 and 0.08 respectively).

**Relapse incidence: **No significant difference was found in the cumulative incidence of relapse between patients who developed CMV infection and those who didn’t after transplantation (P= 0.94; Figure 1c). 

**Figure 1:**
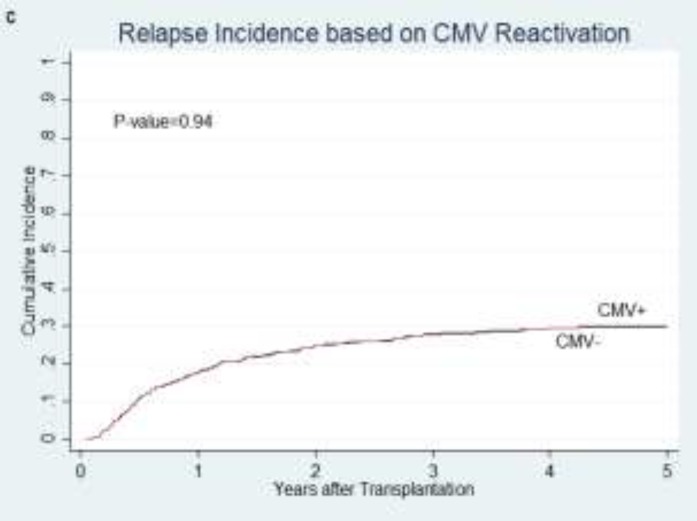
cumulative relapse incidence of all patients in relation to CMV reactivation (c)

R-D- group didn’t show a significant higher relapse compared to other groups (P=0.10). In seropositive and seronegative recipients, serostatus of donor did not affect relapse (P=0.46 and P=0.78). Relapse incidence was lower in pre-transplant serostatus high-risk group though it was not significant (P=0.19).Reactivation of CMV before and after days+30 and +100 were not statistically associated with relapse (P= 0.84 and P=0.45, respectively).In subgroup analysis of patients with AML, relapse rate did not change by CMV reactivation (P=0.58).

**NRM incidence**: The cumulative incidence of NRM was not significantly different between patients with and without CMV reactivation (P= 0.08; Figure 1d).

**Figure 1:**
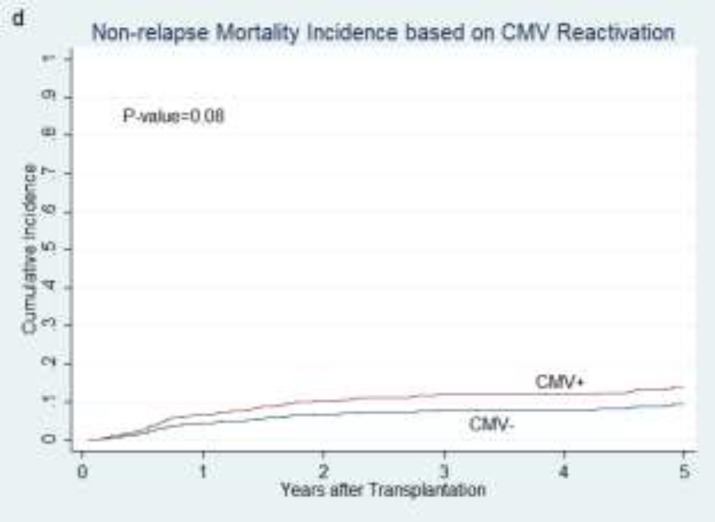
and cumulative non-relapse mortality incidence in relation to CMV reactivation (d).

NRM was not statistically different between pre-transplant risk groups (P=0.69). CMV status of donor and recipient was not associated with NRM (P=0.4 and P=0.49 respectively). NRM was not different between R+D- and R+D+ groups (P=0.68) and also between R-D- and R-D+ patients (P=0.77) as well. Patients from R-D- group did not show any difference in NRM compared with other settings (P=0.45). Reactivation of CMV before and after days+30 and +100 was not statistically associated with NRM (P= 0.37 and 0.78, respectively).

**GVHD:** CMV infection and cGvHD were associated with each other (P=0.004). This relation was also found between CMV infection and aGvHD (P<0.0001). As expected, CMV infection was found more in patients with cGvHD and aGvHD. Pre- transplant serostatus risk group was not affected cGvHD and aGvHD incidence (P=0.52 and P=0.97, respectively). There was no statistically association between occurrence of aGvHD and cGvHD as well as states of R+D+ and R+D- (p=0.85 and P=0.68, respectively). Although not statistically meaningful, aGvHD was found less in R-D- group (P=0.86). Moreover, aGvHD and cGvHD occurrence were not different between R-D- and R-D+ (P=0.07 and P=0.51, respectively). The comparison of R-D- with other groups showed no difference in the prevalence of aGvHD and cGvHD (P=0.87 and P=0.30).

**Univariate and multivariate analysis**

Univariate analyses are shown in Tables 1 and 2. As we declare later in statistical analyses section, all variables with a P-value at or below 0.2 in the univariate Cox proportional hazard regression and the univariate Fine-Gray proportional hazard regression were included in the corresponding multivariate analyses.

Multivariate analysis using the cox regression of OS indicated that CMV infection decreased overall survival (P=0.04, HR: 1.43, 95%CI: 1.00-2.05). Although pre-transplant serostatus high-risk group had a higher survival rate, it was not significant (P=0.06). Female to male donation decreased survival (P=0.04, HR: 1.49, 95%CI: 1.00-2.21). Limited and extensive cGvHD increased survival (P=0.02, HR: 0.59, 95%CI: 0.37-0.94 and P<0.0001, HR: 0.37, 95%CI: 0.25-0.55, respectively).AML patients had better survival than ALL ones (P=0.001, HR: 0.53 95%CI: 0.37-0.77). Donor type influenced OS (P=0.02, HR: 0.35, 95%CI: 0.14-0.87) as shown in Table 1.

**Table 1:** Uni- and Multivariate Cox Proportional Hazard Regression Analyses for OS & RFS

		**OS**				**RFS**			
		**Univariate Cox**		**Multivariate Cox**		**Univariate Cox**		**Univariate Cox**	
		**HR** **(95% CI)**	**p**	**HR** **(95% CI)**	**p**	**HR** **(95% CI)**	**p**	**HR** **(95% CI)**	**p**
Age		0.99 (0.98-1.00)	0.18	1.00 (0.99-1.02)	0.46	0.99 (0.98-1.00)	0.056	1.00 (0.99-1.02)	0.48
Sex Matching	Sex matched	1(Ref.)		1(Ref.)		1(Ref.)		1(Ref.)	
	Male to female	0.77 (0.54-1.11)	0.16	0.90 (0.59-1.38)	0.64	0.70 (0.51-0.97)	0.03	.75 (0.51-1.10)	0.14
	Female to male	1.12 (0.82-1.54)	0.46	1.49 (1.00-2.21)	0.04	1.04 (0.79-1.39)	0.77	1.25 (0.89-1.76)	0.20
cGvHD	No	1(Ref.)		1(Ref.)		1(Ref.)		1(Ref.)	
	Limited	0.66 (0.45-0.98)	0.039	0.59 (0.37-0.94)	0.02	0.69 (0.49-0.97)	0.034	0.60 (0.40-0.90)	0.01
	Extensive	0.46 (0.34-0.63)	0.000	0.37 (0.25-0.55)	0.00	0.46 (0.34-0.60)	0.000	0.42 (0.30-0.59)	0.000
CMV Risk	Low	1(Ref.)		1(Ref.)		1(Ref.)		1(Ref.)	
	intermediate	1.04 (0.43-2.51)	0.93	1.41 (0.56-3.54)	0.46	0.99 (0.43-2.27)	0.975	1.07 (0.46-2.52)	0.87
	High	0.54 (0.33-0.90)	0.02	.61 (0.36-1.03)	0.06	0.60 (0.37-0.97)	0.038	0.61 (0.37-1.02)	0.06
Primary Disease	ALL	1(Ref.)		1(Ref.)		1(Ref.)		1(Ref.)	
	AML	0.53 (0.40-0.70)	0.000	0.53 (0.37-0.77)	0.001	0.57 (0.45-0.72)	0.000	0.57 (0.41-0.78)	0.000
CMV	No	1(Ref.)		1(Ref.)		1(Ref.)			
	Yes	1.22 (0.92-1.63)	0.16	1.43 (1.00-2.05)	0.048	1.16 (0.90-1.5)	0.25		
aGvHD	No & I	1(Ref.)		1(Ref.)		1(Ref.)			
	II & III	1.24 (0.94-1.62)	0.12	1.27 (0.85-1.71)	0.29	1.04 (.82-1.33)	0.71		
Donor Type	Full Matched Sibling	1(Ref.)		1(Ref.)		1(Ref.)			
	Others	0.55 (0.26-1.18)	0.12	0.35 (0.14-0.87)	0.023	0.75 (0.42-1.34)	0.327		

The proportional hazards assumption was confirmed (all Ps>0.05). OS: Overall Survival; RFS: Relapse-Free Survival; HR: Hazard Ratio

RFS was not associated with CMV reactivation in univariate analysis, so CMV infection was excluded from multivariate analysis of RFS. Multivariate analysis of RFS among pre-transplant risk groups showed that patients of high-risk group had higher RFS, which was not statistically significant (P=0.06). Different sex settings of donation did not affect RFS as well (P=0.14 and P=0.20). Limited and extensive cGvHD resulted in better RFS (P=0.01, HR: 0.60, 95% CI: 0.40-0.90 and P<0.0001, HR: 0.42, 95%CI: 0.30-0.59, respectively). RFS was better in AML (P<0.0001, HR: 0.57, 95%CI: 0.41-0.78) as shown in Table 1. 

Multivariate analysis of relapse incidence showed that pre-transplant high-risk group had less relapse incidence than other groups (P=0.012, HR: 0.61, 95%CI: 0.41-0.89).There was less relapse incidence in male to female donation as well (P=0.01, HR: 0.67, 95%CI: 0.48-0.93). Relapse incidence was lower in both limited and extensive cGvHD (P=0.007, HR: 0.64, 95%CI: 0.47-0.89 and P<0.0001, HR: 0.31, 95%CI: 0.23-0.41, respectively). AML patients had lower relapse incidence than ALL ones, too (P<0.0001, HR: 0.45, 95%CI: 0.34-0.59) as shown in Table 2.

**Table 2:** Uni- and Multivariate Fine&GrayCompetingRiskRegressionAnalyses for Relapse and NRM

		**Relapse**						**NRM**					
		**Univariate competing ** **risk regression**			**Multivariate competing ** **risk regression**			**Univariate competing risk ** **regression**			**Multivariate competing ** **risk regression**		
		**SHR ** **(95% CI)**	**p**		**SHR ** **(95% CI)**	**p**		**SHR** **(95% CI)**	**p**		**SHR** **(95% CI)**	**p**	
Age		0.98 (0.97-0.99)		0.001	1.00 (0.99-1.01)		0.19	1.00 (0.99-1.02)		0.460			
Sex Matching	Sex matched	1(Ref.)			1(Ref.)			1(Ref.)			1(Ref.)		
	Male to female	0.64 (0.49-0.83)		0.001	0.67 (0.48-0.93)		0.019	1.14 (0.70-1.84)		0.598	1.30 (0.80-2.11)		0.29
	Female to male	0.75 (0.58-0.96)		0.023	0.90 (0.66-1.22)		0.514	2.48 (1.68-3.67)		0.000	2.59 (1.70-3.95)		0.000
cGvHD	No	1(Ref.)			1(Ref.)			1(Ref.)			1(Ref.)		
	Limited	0.66 (0.51-0.86)		0.002	0.64 (0.47-0.89)		0.007	0.92 (0.47-1.81)		0.812	0.89 (0.45-1.76)		0.742
	Extensive	0.30 (0.24-0.39)		0.000	0.31 (0.23-0.41)		0.000	2.04 (1.28-3.27)		0.003	1.70 (1.04-2.78)		0.034
CMV Risk	Low	1(Ref.)			1(Ref.)			1(Ref.)					
	intermediate	.83 (0.40-1.74)		0.63	0.85 (0.43-1.69)		0.639	0.92 (0.28-3.01)		0.886			
	High	0.59 (0.39-0.89)		0.012	0.61 (0.41-0.89)		0.012	0.66 (.35-1.26)		0.215			
Primary Disease	ALL	1(Ref.)			1(Ref.)			1 (Ref.)					
	AML	0.46 (0.38-0.57)		0.000	0.45 (0.34-0.59)		0.000	1.12 (0.79-1.6)		0.520			
CMV	No	1(Ref.)						1 (Ref.)			1(Ref.)		
	Yes	1.00 (0.81-1.25)		0.94				1.54 (1.09-2.20)		0.015	1.62 (1.11-2.38)		0.013
aGvHD	No & I	1(Ref.)			1(Ref.)			1 (Ref.)			1(Ref.)		
	II & III	0.87 (0.71-1.06)		0.166	0.88 (0.69-1.14)		0.350	1.74 (1.23-2.48)		0.002	1.29 (.88-1.90)		0.19
Donor Type	Full Matched Sibling	1(Ref.)						1 (Ref.)					
	Others	0.84 (0.54-1.31)		0.455				0.52 (0.19-1.42)		0.203			

NRM, Non-Relapse Mortality; SHR, Sub-Distribution Hazard Ratio

Multivariate analysis of NRM showed that patients who developed CMV reactivation had higher NRM rate (P=0.01, HR: 1.62, 95%CI: 1.11-2.38). Female to male transplantation increased NRM (P<0.0001, HR: 2.59, 95%CI: 1.70-3.95). Moreover, extensive cGvHD in patients resulted in higher NRM (P=0.034, HR:1.70, 95%CI: 1.04-2.78),but limited cGvHD and aGvHD had no effect on NRM(P=0.74 and p=0.19 respectively) as displayed in Table2.

**Figure 2:**
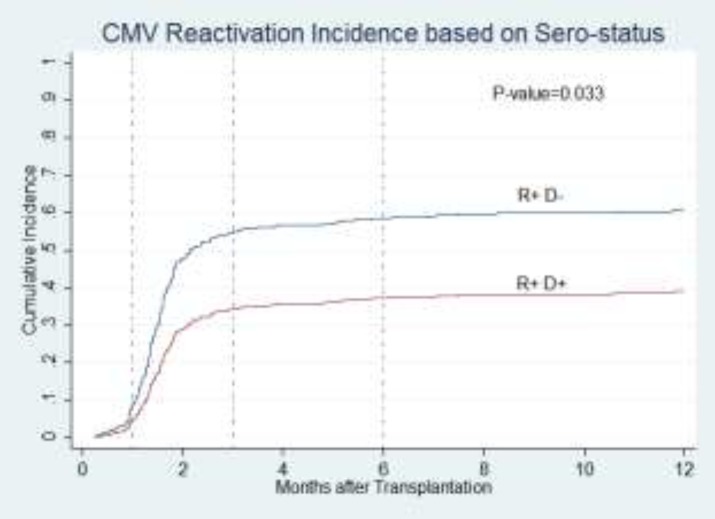
Cumulative incidence probabilities of CMV reactivation according to sero-status of recipients and donors.

Another important finding was that CMV reactivation incidence was different between positive recipients grafted from seronegative donors and those grafted fromseropositive donors (P=0.033, Figure 2).

Since there were only 45 patients treated with transplantation from non-identical sibling donors, we also analyzed data for the rest 660 identical fully- matched sibling transplantations. CMV infection between R+D+ and R+D- was not statistically different (P=0.12). Also, CMV reactivation among CMV serostatus risk groups did not show any difference. But CMV infection was associated with aGvHD and cGvHD (P<0.0001 and 0.006). Considering CMV infection and other variables, multivariate cox regression of OS for this group was as same asthe whole patients,but, in this group, female to male donation did not change OS.

Finally, Multivariate analysis of RFS for this group showed that male to female donation improved RFS (P=0.03, HR: 0.70, 95%CI: 0.50-0.97). Both limited and extensive cGvHD made better RFS (P=0.03, HR: 0.68, 95%CI: 0.47-0.97 and P<0.0001, HR: 0.42, 95% CI: 0.31-0. 57, respectively).

AML patients had better RFS than ALL ones (P<0.0001, HR: 0.57, 95%:0.44-0.75). CMV reactivated patients had worse RFS on the other hand (P=0.002, HR: 1.51, 95%CI:1.15-1.99). Multivariate analysis of relapse and NRM incidence in identical full-matched sibling group showed an increase in NRM among patients with CMV reactivation which is similar to results of the whole patients. In terms of other variables, results were the same.

## Discussion

 The relationship between CMV infection after HSCT and its impact on transplantation outcomes has been a subject of debate. In this study, we evaluated CMV serostatus in donors and recipients of HSCT and their effects on CMV reactivation incidence in patients. Transplantation outcomes such as OS, RFS, relapse and NRM regarding pre-transplant CMV serostatus and CMV reactivation were also studied. The relevance between CMV infection and acute as well as chronic GvHD were also examined. Since in the setting of a transplant recipient CMV serostatus is not changeable, choosing an appropriate donor could help us achieve better results. To evaluate effect of donor CMV serostatus on outcomes, we decided to study donor-recipient pairs in three risk groups: donors with positive serology for CMV were accrued to high-risk group including either R+ or R-, seronegative donors were studied in low-risk group which was composed of patients who were supposed to have the minimum chance to present with CMV infection(R-D-) and the rest of patients were categorized as intermediate group(R+D-). We found no statistically difference in CMV reactivation among these risk groups. Our analysis revealed that CMV reactivation happened less frequently in seropositive recipients grafted from seropositive donors compared to transplantation from seronegative donors(R+D-). Hirszfeld Institute of Immunology and Experimental Therapy study reported that R+D- transplantations suffered more frequently from CMV infection after HSCT as compared to those grafted from other donor-recipient serostatusmatching^23^. These two study results are not exactly the same, but both concluded that CMV would present less in R+D+ groups than R+D- matchings.As mentioned in our results, in seronegative recipients, donor seropositivity did not influence CMV reactivation.

Our analysis showed that patients who developed CMV infection had lower OS and higher non-relapse mortality rate (NRM) with no change in RFS and relapse rate. These results confirm Takenaka study which showed an increase in non-relapse mortality and overall mortality in patients with CMV reactivation^24^. Same results on decrease in OS among CMV reactivated patients were reported in Transplant Infectious Disease journal^25^. In contrast, Green et al. study reported no association between CMV reactivation and overall mortality^26^, however, they also confirmed an increase in NRM with CMV reactivation. Since there were only 45 patients with transplantation from non-identical sibling donors, we analyzed outcomes in multivariate cox regression for the rest 660 identical full-matched sibling transplantations. OS in this group was also decreased in case of CMV reactivation. In contrast to results of CMV reactivation in the whole patients that showed no effect on RFS, analysis of fully matched identical sibling revealed decrease in RFS.

In our study, OS and RFS were better in high-risk pre-transplant group(R+D+ and R-D+), but these differences were not significant. In a study in the registry of the ALWP of EBMT, donor CMV seropositivity and/or recipient seropositivity in comparison to R-D- was associated with a significant decrease in RFS and OS^26^. We also compared CMV infection and outcomes between low-risk group(R-D-) and other groups. OS and RFS of seronegative recipients with graft from seronagtive donors were lower than other patients. Since CMV infection prevalence is relatively high in our country, the small number of patients included in R-D- group might make our results statistically not interpretable and it could explain the opposite results of two studies. Relapse multivariate analysis indicated that CMV reactivation did not change relapse rate although transplantations from positive donors (high-risk group) resulted lower relapse. Elmaaglaci et al. reported an independent reduction of relapse risk in leukemia after early CMV infection in adult AML patients^3^. In our data, time to CMV reactivation, either 30 or 100 days after transplantation, was not associated with relapse rate. In subgroup analysis, we did not find any difference in relapse rate of AML patients with CMV reactivation. Another study reported a beneficial effect of CMV infection on relapse in AML patient, but no other hematological malignancies included in the study^24^. Green study showed that CMV infection decreased relapse within 100 days after transplantation only in AML patients^26^,but early CMV reactivation did not affect relapse one year after transplantation in any primary diseases.

As expected, CMV infection significantly occurred more frequently in patients with aGvHD and cGvHD that it could be the result of using immunosuppressive therapy in these patients. In our study, both acute and chronic GvHDprevalence were not different between pre-transplant risk groups. In comparison of R-D-patients with other ones, we didn’t find any difference in incidence of either acute or chronic GvHD. Schmidt-Hieber et al. study also did not report a significant difference in the incidence of aGvHD and cGvHDby the comparison of R-D- with R+ and/or D+ groups^26^.Finally, in addition to the univariate analyses of the effects of CMV on outcomes, multivariate cox regression revealed that both limited and extensive cGvHD and also AML patients compared to ALL were associated with better OS and RFS results. Extensive cGvHD and female to male donation increased NRM. Relapse rate decreased in AML patients, male to female donation and cGvHD.

## CONCLUSION

 Our results indicate that CMV reactivation was increased in R+D- patients as compared to R+D+ ones. On the other hand, we showed that CMV infection resulted in lower OS and higher NRM without any effect on relapse and RFS. In contrast to general belief and practice, it could be suggested that when there is a seropositive recipient, seropositive donor is preferred to a seronegative one. Considering less relapse rate in donor positive transplantations, regardless of recipient CMV serostatus and CMV reactivation, choosing seropositive donor is highly recommended.
